# Pro-inflammatory mediators expression by pulp cells following tooth whitening on restored enamel surface

**DOI:** 10.1590/0103-6440202204688

**Published:** 2022-04-29

**Authors:** Diana Gabriela Soares, Nancy Tomoko Sacono, Ana Paula Dias Ribeiro, Maria Luisa Leite, Carla Caroline de Oliveira Duque, Marjorie de Oliveira Gallinari, Leandro Edgar Pacheco, Josimeri Hebling, Carlos Alberto de Souza Costa

**Affiliations:** 1 Department of Operative Dentistry, Endodontics and Dental Materials, Bauru School of Dentistry, University of São Paulo, Bauru, SP, Brazil; 2 Department of Pediatric Dentistry, Unip - Universidade Paulista, Goiania Goias, Brazil, Br 153 Highway, Km 503, area 1-5, s/n - Fazenda Botafogo, , GO, 74845-090, Brazi; 3 Department of Restorative Dental Sciences, College of Dentistry, University of Florida, 1395 Center Drive, 100405, Gainesville, FL, 32606, USA.; 4 Department of Dental Materials and Prosthodontics, Araraquara School of Dentistry, Universidade Estadual Paulista, Araraquara, Brazil.; 5 Department of Orthodontics and Pediatric Dentistry, Araraquara School of Dentistry, São Paulo State University, Araraquara, Brazil.; 6 Department of Physiology and Pathology, São Paulo State University (UNESP), School of Dentistry, Araraquara, Brazil

**Keywords:** Tooth-bleaching, odontoblasts, inflammation, toxicity, adhesion

## Abstract

This paper aimed to assess the influence of adhesive restoration interface on the diffusion of hydrogen peroxide (H_2_O_2_), indirect toxicity, and pro-inflammatory mediators expression by odontoblast-like cells, after in-office tooth whitening. Dental cavities prepared in bovine enamel/dentin discs were adhesively restored and subjected or not to hydrolytic degradation (HD). A whitening gel with 35% H_2_O_2_ (WG) was applied for 45 min onto restored and non-restored specimens adapted to artificial pulp chambers giving rise to the groups: SD- intact discs (control); SD/HP- whitened intact discs; RT/HP- restored and whitened discs; and RT/HD/HP- restored and whitened discs subjected to HD. The extracts (culture medium + WG components diffused through enamel/dentin/restoration interface) were collected and applied to odontoblast-like MDPC-23 cells. The study evaluated the amount of H_2_O_2_ in the extracts, as well as the cell viability (CV), cell morphology (CM), and gene expression of inflammatory mediators (TNF-α and COX-2) by the pulp cells exposed to the extracts (ANOVA and Tukey tests; 5% significance). All whitened groups presented lower CV than SD (control; p<0.05). The highest CV reduction and gene expression of TNF-α and COX-2 was observed in the RT/HD/HP group in comparison with SD/HP and RT/HP (control; p<0.05). CM alterations occurred in all whitened groups. The intensity of these cell side effects was directly related with the amount of H_2_O_2_ in the extracts. We concluded that adhesive restoration of dental cavity increases the H_2_O_2_ diffusion after in-office whitening, enhancing the indirect toxicity of this therapy and trigger pro-inflammatory overexpression by MDPC-23 cells.

## Resumo

Este trabalho teve como objetivo avaliar a influência da interface de uma restauração adesiva na difusão do peróxido de hidrogênio (H_2_O_2_), toxicidade indireta e expressão de mediadores pró-inflamatórios por células odontoblastóides, após clareamento dental em consultório. Cavidades dentárias preparadas em discos de esmalte / dentina foram restauradas com adesivo e submetidas ou não à degradação hidrolítica (HD). Um gel clareador com 35% H_2_O_2_ (WG) foi aplicado por 45 min em discos restaurados e não restaurados adaptados às câmaras pulpares artificiais dando origem aos grupos: SD- discos intactos (controle); SD / HP - Discos intactos clareados; RT / HP - discos restaurados e clareados; e RT / HD / HP - discos restaurados, clareados e submetidos a HD. Os extratos (meio de cultura + componentes WG difundidos através da interface esmalte/dentina/restauração) foram coletados e aplicados em células odontoblastóides MDPC-23. Foi avaliada a quantidade de H_2_O_2_ nos extratos, bem como a viabilidade (CV), morfologia (CM) e expressão gênica de mediadores inflamatórios (TNF-α e COX-2) pelas células pulpares expostas aos extratos (ANOVA e testes de Tukey; 5% de significância). Todos os grupos clareados apresentaram menor CV do que SD (controle; p <0,05). A maior redução CV e expressão gênica de TNF-α e COX-2 foi observada no grupo RT / HD / HP em comparação com SD / HP e RT / HP (controle; p <0,05). Alterações na CM ocorreram em todos os grupos clareados. A intensidade desses efeitos celulares teve relação direta com a quantidade de H_2_O_2_ nos extratos. Concluímos que a presença de uma cavidade contendo restauração adesiva aumenta a difusão de H_2_O_2_ após o clareamento em consultório, o que, por sua vez, aumenta a toxicidade indireta dessa terapia e desencadeia a expressão de mediadores pró-inflamatórios pelas células pulpares MDPC-23.

## Introduction

Post tooth whitening sensitivity (PTWS) has been strongly correlated with the diffusion of H_2_O_2_ through enamel and dentin reaching the pulp chamber ^1^. Previous laboratory investigations showed that this reactive oxygen-derived species (ROS) interact with pulp cells to cause oxidative stress, lipidic peroxidation, and even direct cell death by necrosis ^2^. In vivo studies have already proved that different in-office whitening protocols cause pulp inflammation featured by exudative vascular phenomena, infiltration of neutrophils, macrophages and mast cells ^3^. The release of biochemical mediators bradykinin, histamine and prostaglandins, along with neuronal receptors activation and peptide neurotransmitter expression in the inflamed pulp tissue, plays a crucial role on PTWS ^4^. In addition to pulp cells toxicity ^5^, several in vitro studies have demonstrated the overexpression of pro-inflammatory mediators (IL-6, IL-1β, TNF-α, and COX-2) by pulp cells exposed even to low concentrations of H_2_O_2_ ^5^. 

Clinical trials have shown that PTWS is intensified after whitening adhesively-restored anterior teeth using gels with high concentration of H_2_O_2_ ^6^. This side effect has been correlated with the high inward diffusion of such ROS through the tooth/restoration interface ^7^. It has been demonstrated that the sensitivity of tooth/restoration interface to H_2_O_2_ is greatly influenced by the restorative material due to the different degrees of interface degradation over time ^8^. In a previous study, Soares et al. ^9^ described that fresh and non-degraded tooth/restoration interface provides enough seal against H_2_O_2_ diffusion. However, the adhesive system category and its resistance against degradation influence the H_2_O_2_ diffusion, preventing or not the adverse effects caused by tooth whitening therapy on pulp cells ^7^. Since in the clinical scenario a preexistent restoration often has no background information, the application of whitening gel on adhesively restored teeth may be considered a challenge from a biological point of view ^6^.

Therefore, the present study aimed to assess the influence of adhesive restoration interface on the diffusion of H_2_O_2_, indirect toxicity, and pro-inflammatory mediators expression by odontoblast-like cells, after in-office tooth whitening. For this purpose, restored and non-restored cavities prepared in enamel/dentin discs subjected or not to hydrolytic degradation were adapted to artificial pulp chambers. Then, the amount of H_2_O_2_ capable of diffusing across enamel/dentin and/or tooth/restoration interface was determined and its potential to cause cytotoxicity was evaluated. Based on the fact that odontoblast layer is the first pulp cell line that orchestrate the inflammatory cascade of tissue response ^10^, a monolayer of MDPC-23 odontoblast-like cells exposed to H_2_O_2_ was used to assess the expression of pro-inflammatory mediators following in-office tooth whitening therapy. The hypothesis of this work is that the tooth/restoration interface and its degradation over time increase the H_2_O_2_ diffusion, causing toxic effects and inducing the overexpression of pro-inflammatory mediators by pulp cells. 

## Material and methods

### Enamel/Dentin Discs

Eighty-eight enamel/dentin discs with 3.5-mm thickness and 5.6-mm diameter were obtained from the buccal surface of intact bovine incisors. Standardized cavities (1.6-mm diameter and 2.5-mm deep) were prepared in forty-four of these discs, as described by Soares et al. 2016 ^9^. The cavities were restored with one-step self-etching adhesive system (iBond SE Plus - Heraeus Kulzer, Germany) and composite resin (Filtek™ Z350 - 3M ESPE, Saint Paul, Minnesota, USA), as follows: one layer of iBond SE Plus was applied on the dentin surface under friction for 20 seconds, followed by 10 seconds of gentle air-drying. This procedure was repeated one more time, and the bonding agent was light-cured for 20 seconds (450 mW/cm^2^ Curing Light XL 300, 3M ESPE). Then, two increments of a nanofilled composite resin (Filtek™ Z350 - 3M ESPE) were individually inserted in the cavities, and each increment was light-cured for 20 seconds. Twenty-four hours after cavity restoration, the resin surface was polished with sequential Soflex discs (Sof-Lex Pop On - 3M ESPE) at low speed. The enamel surface of the intact enamel/dentin discs (SD) received a round resin coating with 1.6-mm diameter, to standardize the enamel area to be exposed to the whitening gel. For this purpose, the specific area of enamel surface was etched with 37% phosphoric acid for 30 sec, followed by application of two layers of bonding agent (light-cured for 20 sec.). Finally, a layer of composite resin (1-mm thick) was applied and also light-cured for 20 sec.

**Figure 1:**
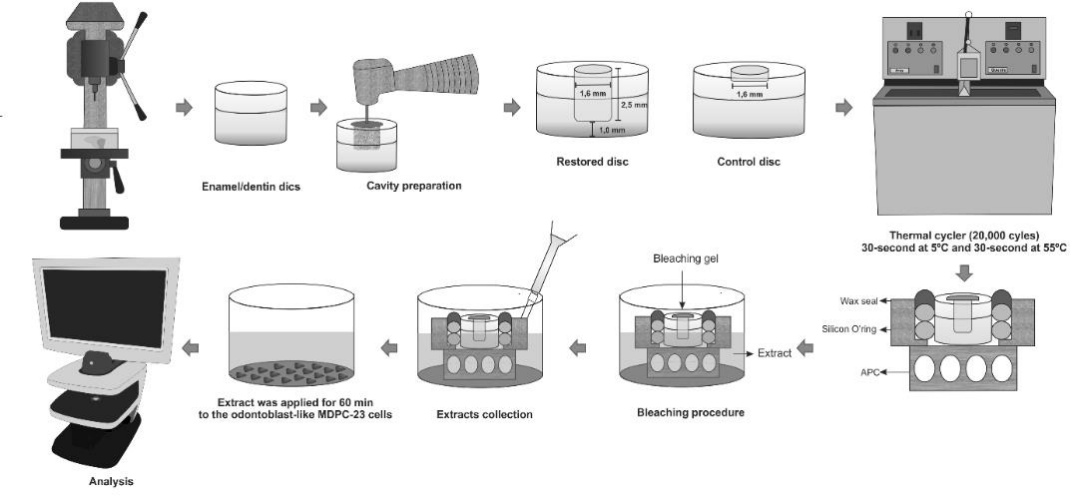
Flowchart representing the methodology

The enamel/dentin discs with adhesive-restored cavities (RT) were subjected or not to hydrolytic degradation (HD). This procedure included thermocycling in a thermal cycler (MSCT-3 plus; Marcelo Nucci-ME, São Carlos, SP, Brazil) totalizing 20,000 cycles at 5 and 55°C with a 30-second well time in each bath, followed by storage in thymol 0.1% at 37^o^C for 6 months (thymol solution was replaced every week). The dentin surface of discs was treated with EDTA 0.5 N for 30 seconds for smear layer removal. The discs were individually adapted to artificial pulp chambers (APCs) as described by Soares et al. (2014) ^11^. Two silicon o’rings (Rodimar Rolamentos Ltda, Araraquara, SP, Brazil) were used to adapt the disc in the CPA, and warm wax was employed to seal the disc edge. The APCs with the enamel/dentin discs in position were sterilized in ethylene oxide gas. The following groups were established (n=22): SD- intact discs (control); SD/HP- whitened intact discs; RT/HP- restored and whitened discs; and RT/HD/HP- restored discs subjected to HD and then whitened (Figure 1). 

### Experimental procedure

The APC/disc set was individually placed into wells of sterilized 24-well plates containing 1 mL of Dulbecco’s Modified Eagle Medium (DMEM; Gibco, Grand Island, NY, USA), supplemented with 100 IU/mL penicillin, 100 (g/mL streptomycin, 2 mmol/L glutamine (Gibco). The dentin surface of the discs was maintained in contact with the DMEM and the enamel surface remained exposed to receive the whitening gel. The in-office tooth whitening therapy was performed applying a gel with 35% H_2_O_2_ (Whiteness HP 35%; FGM, Joinville, SC, Brazil) for 45 min. (3x15 min.) on enamel. The amount of bleaching gel was standardized in 40 µL by means of a pipette coupled with a capillary piston tip (Microman E, Gilson, Middelton, WI, USA). Then, the extract (DMEM + components of the whitening gel that reached the pulpal space of the CPA) was collected and immediately applied for 60 min. to the previously cultured odontoblast-like MDPC-23 cells (60,000 cells/well) for 48 h in wells of 24-well plates (Figure 1). Then the cell viability and morphology, as well as the expression of inflammatory mediators, were assessed. The quantification of H_2_O_2_ in the extracts was performed in the same specimens used for MTT assay. Sample size was based on previous investigations from our group ^2^ in which sample calculation was performed with DDS Research (Sample Size Calculator, average, two samples, a=5%; b=95%), and six to eight samples per group were stablished for each quantitative assay.

### Cell viability assay (n=8)

Immediately after incubating for 60 min. the MDPC-23 cells in contact with the extracts obtained for each group, the extracts were aspirated and the MTT solution (5 mg/mL, Sigma-Aldrich, St. Louis, MO, USA) at 1:10 was applied to the cells for 4 hours (37^o^C and 5% CO_2_). Thereafter, formazan crystals were dissolved in acidic isopropanol (Sigma-Aldrich, St. Louis, MO, USA) and the absorbance was read at 570 nm (Synergy H1, BioTek, Winooski, VT, USA). The absorbance value of control and experimental groups was transformed into percentage by mean value of the negative control group (SD). 

### Cell morphology (n=2)

For this analysis, the MDPC-23 cells were seeded on sterilized round-shaped glass coverslips (13-mm diameter) previously placed on the bottom of wells of 24-well plates. Then, the same experimental procedure was performed as described above. Immediately after exposing the cells to the extracts for 60 min., the cells were fixed in 2.5% buffered glutaraldehyde solution isopropanol (Sigma-Aldrich), followed by post-fixation in 1% osmium tetroxide (Sigma-Aldrich). After rinsing the cells with phosphate buffer solution (PBS), the dehydration protocol of cells was carried out using a sequence of increasing ethanol concentrations (30, 50, 70, and 100%) and 1,1,1,3,3,3-hexamethyldisilazane (HMDS; Sigma-Aldrich). Then, the coverslips with the MDPC-23 cells attached on them were removed from the wells and mounted on metallic stubs, which were kept in desiccator for 72 h. After sputter-coating the coverslips with gold, the morphology of cells that remained attached to the substrate was assessed by scanning electron microscopy (Philips FEG XL 30; Oxford Instruments, Inc., Concord, MA, USA).

### Quantification of HP Diffusion (n=6)

A 100 µL aliquot of the extract of each group was transferred to tubes containing 900 (L of acetate buffer solution (2 mol/L, pH 4.5). Then, a 100 µL quantity of PBS plus extract was transferred to experimental tubes to react with leucocrystal violet (0.5 mg/mL; Sigma-Aldrich) and horseradish peroxidase enzyme (1 mg/mL; Sigma-Aldrich). The final volume of reaction was adjusted to 3 mL with distilled water, and the optical density of solutions was measured at 600-nm wavelength (Synergy H1, BioTek). A standard curve was used for conversion of the optical density obtained in the specimens into µg/mL of H_2_O_2_. 

### Real-time PCR (n=6)

Following the aspiration of extracts that remained in contact with the MDPC-23 cells for 60 min., 1 mL of complete DMEM was applied in each well. After 6 h incubation, the total ribonucleic acid (RNA) was extracted. This 6 h period was chosen based on a time-course analysis to determine the ideal time-point to observe the gene expression (data not shown). Total RNA was extracted with Trizol reagent, as previously described ^12^. One microgram of total RNA, following DNase I treatment, was reverse-transcribed into single-stranded cDNA with a HighCapacity cDNA Reverse Transcription Kit (Applied Biosystems, Foster City, CA, USA), according to the recommended protocol [25^o^C (10 min), 37^o^C (120 min), 85^o^C (5s), 4^o^C] ^12^. For relative quantification of inflammatory mediators, the following Syber Green primers (Sigma-Aldrich) were used: IL1-( (F 5′-AAAGCCTCGTCGTGTCGG -3′; R 5′-CCTTTGAGGCCCAAGGGC-3′), IL-6 (F 5′-GAGGATACCACTCCCAACAGACC-3′; R 5′-AAGTGCATCATCGTTGTTCATACA-3′), TNF-α (F 5′-CCCTCCTGGCCAACGGCA-3′; R 5′-TCGGGGCAGCCTTGTCCC-3′), COX-2 (F 5′-ACCCTGCCTACGAAGGAACT-3′; R 5′-ACCACGGTTTTGACATGGGT-3′) and (-actin (F 5′-GGACCTGACGGACTACCTCATG-3′; R 5′-TCTTTGATGTCACGCACGATTT-3′). Amplification assays were performed with Applied Biosystems Master Mix, and fluorescence was determined with StepOne Plus equipment (Applied Biosystems). The CT values for each specimen were normalized by an endogenous control gene ((-actin). Thereafter, the mean CT value of the SD group was used to normalize the CT value of both control and experimental groups.

### Statistical Analysis

Two independent experiments were performed. Data were compiled and analyzed by Kolmogorov-Smirnov and Levene tests. Since normal data were obtained, one-way ANOVA and Tukey’s test were used for observation of the significant differences between the study groups, for the quantitative data obtained in cell viability, H_2_O_2_ diffusion and gene expression. All statistical analyses were carried out at a significance level of 5%.

Two independent experiments were performed for each assay. Data were compiled and analyzed one-way ANOVA and Tukey’s test were used for cell viability, as well as H2O2 diffusion, oxidative stress, DE, DL, Da, and Db analysis. Data of pH measurement were analyzed with repeated measure two-way ANOVA and Dunnet’s test to compare pH values at each time-point with those from HP group. All statistical analyses were carried out at a significance level of 5%.

## Results

### Cell viability and morphology

**Figure 2:**
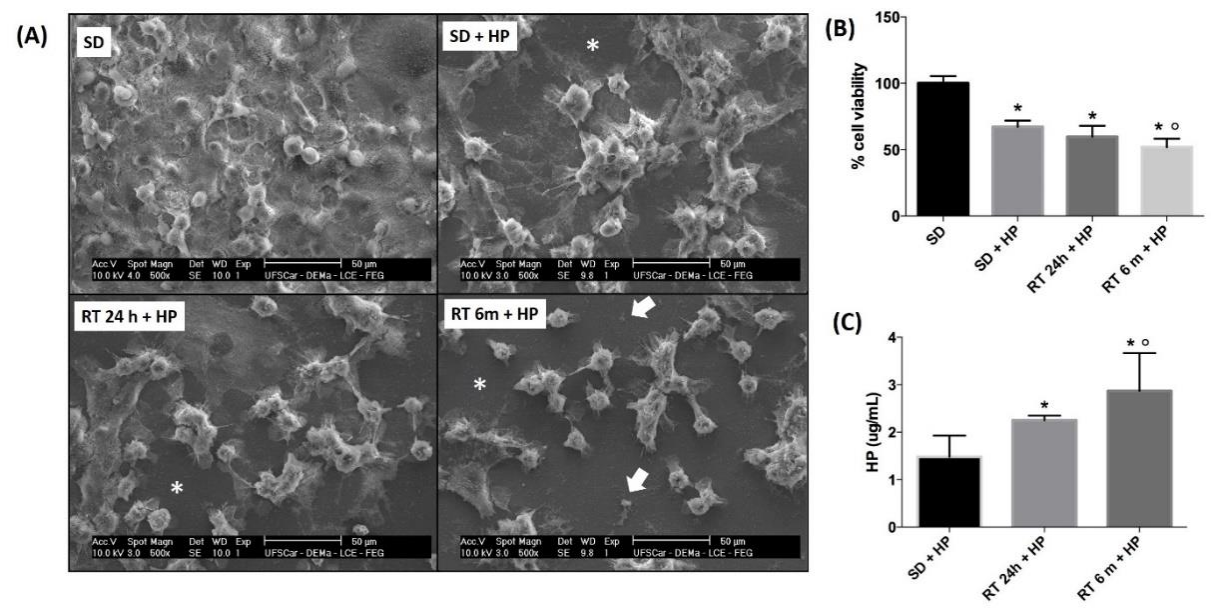
(A) Representative images of SEM analysis, 500X. SD Group 24 hours: The cells exhibit normal morphology and are close to confluence, presenting wide cytoplasm with several cytoplasmic extensions and presence of mitosis. Whitened groups (SD/HP, RT/HP and RT/HD/HP): the whitening agent applied for three consecutive times caused intense morphological changes in MDPC-23 cells. The cells exhibited rounded morphology and remarkable reduction in size, characterized by marked contraction of the cytoplasm. Glass substrate can be observed (*) on bleached groups, as a consequence of morphological alterations and possibly by cell detachment, as cellular remains can be observed on RT 6m + HP (arrow). (B) Mean and standard deviation of cell viability values (%) of the experimental groups (n=6; ANOVA and Tukey tests; p<0.05). (C) Mean and standard deviation of H_2_O_2_ quantification of the experimental groups (n=6; ANOVA and Tukey tests; p<0.05). Asterisk (*) indicates significant difference in relation to the SD/HP group. Open circle (°) indicates significant difference in relation to the RT/HP group

Statistically significant cell viability decreases of 32.9%, 40.3% and 48.2% occurred in the groups SD/HP, RT/HP and RT/HD/HP, respectively, in comparison to SD (control) (p<0.05; Figure 2b). The lowest cell viability values were observed in RT/HD/HP, which was different when compared to SD/HP (p<0.05). In the control group, several MDPC-23 cells exhibiting large membrane and several thin cytoplasmic processes were covering the glass substrate. However, in those groups in which the enamel/dentin discs (with or without adhesively restored cavity) were submitted to in-office whitening protocol, some cells detached from the substrate. The MDPC-23 cells that remained adhered to the round-shaped coverslips exhibited membrane contraction that was more intense in the group RT/HD/HP (Figure 2a).

### Quantification of H_2_O_2_ in the extracts

The highest amount of H_2_O_2_ was found in the extracts from all groups in which the in-office whitening protocol was performed, when compared to the control (p<0.05; Figure 2c). However, the extracts from SD/HP showed lower concentration of H_2_O_2_ than in RT/HP and RT/HD/HP groups (p<0.05). Extracts with the highest H_2_O_2_ concentration were determined in the RT/HD/HP group (p<0.05). 

### Gene expression of pro-inflammatory mediators

**Figure 3:**
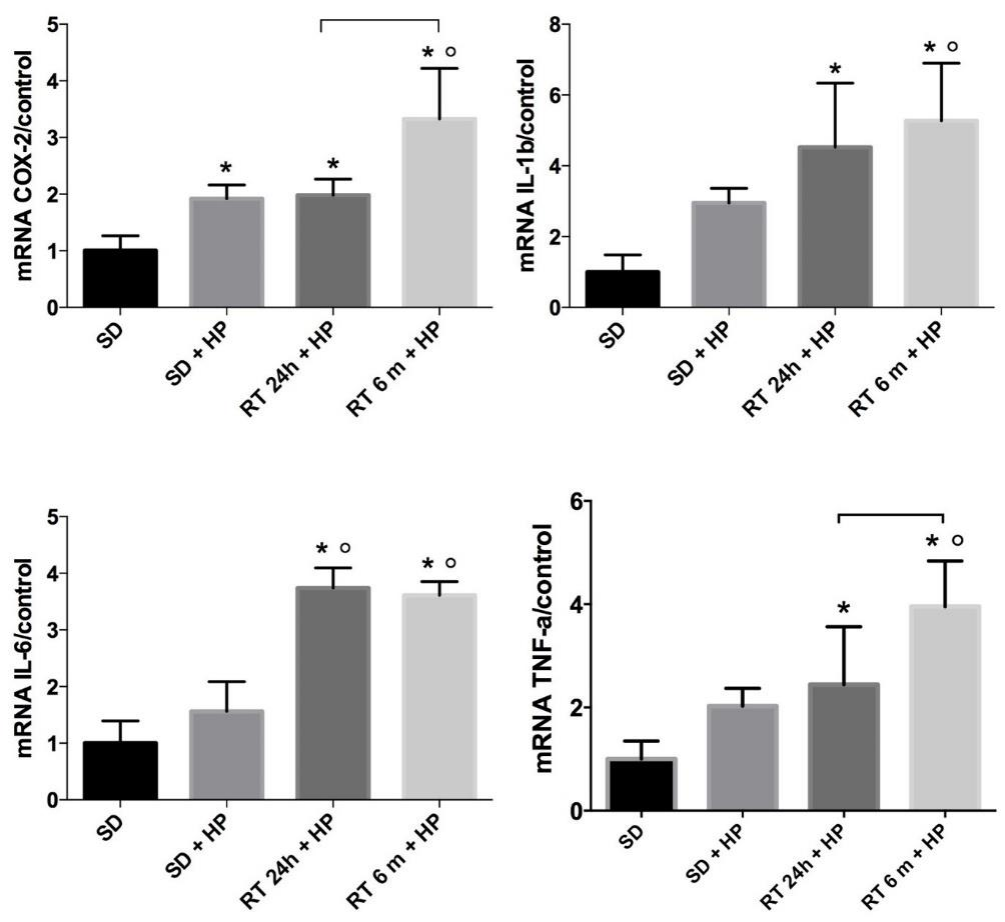
Mean and standard deviation of gene expression values of experimental groups (n=6; ANOVA and Tukey tests; p<0.05). Asterisk (*) indicates significant difference in relation to the SD group. Open circle (°) indicates significant difference in relation to the SD/HP group. [ indicates differences between RT/HP and RT/HD/HP groups

The expression of COX-2 by the MDPC-23 cells was higher in SD/HP, RT/HP and RT/HD/HP in comparison with the control - SD (p<0.05; Figure 3). The highest expression of COX-2 occurred in the RT/HD/HP (p<0.05). The gene expression of IL-1β, IL-6 e TNF-α was higher only in RT/HP and RT/HD/HP groups when compared to SD (p<0.05). Statistically higher IL-1β and TNF-α gene expression by the pulp cells was observed in RT/HD/HP than in SD/HP groups (p<0.05).

## Discussion

According to the results found on this investigation, the hypothesis was partially rejected, as the presence of an adhesive interface prepared with a one-step self-etch adhesive system increased the toxic effects of tooth whitening in vitro only when it was subjected to hydrolytic degradation. In the whitening protocol selected for this study, a gel with 35% H_2_O_2_ was applied on the specimens for 45 min, since this esthetic therapy has been widely used in offices. However, clinical studies have shown that this professional technique causes sensitivity in anterior teeth ^16^, which is worsened in the presence of unsatisfactory adhesive restorations. In vitro studies also demonstrated that in-office tooth whitening causes indirect toxicity to pulp cells, whose intensity is influenced by the presence of adhesive restoration on the surface where the gel is applied ^7^ ^,^ ^11^. Also, microscopic analyses in rat ^3^ and human teeth ^17^ submitted to in-office whitening demonstrated occurrence of extensive pulp damage associated with tissue inflammation of variable intensity.

In the present study, application of the whitening protocol on intact enamel/dentin discs (SD/HP) caused an approximate reduction of 33% in the viability of MDPC-23 cells, which exhibited morphological changes compared to the control. These results corroborate data obtained in previous studies ^11^. In this SD/HP group, cells that remained adhered to the glass substrate after exposure to the extracts showed increase in COX-2 expression. In groups in which the specimens were restored and whitened, the higher concentration of H_2_O_2_ in extracts reduced the cell viability by more than 50%. However, the thermocycling of restored specimens before whitening (RT/HD/HP) caused the most intense cytotoxic effects observed in the present study. These data were corroborated by SEM images, in which a smaller number of MDPC-23 cells, with rounded morphology, remained adhered to the substrate compared to the other groups. These scientific data confirm the hypothesis of the present study.

The most intense adverse effects observed in the RT/HD/HP group may have occurred because specimens were submitted to the thermocycling technique, which aims to simulate the thermal changes occurring in the oral cavity. The one-step SE system iBond SE Plus adhesive system was used in this study to simulate critical conditions regarding establishment of the tooth/resin bonding interface, as the relatively impermeable hydrophobic layer is not used to protect the adhesive interface from water transudation ^13^ ^,^ ^14^ ^,^ ^15^. The thermocycling technique allowed assessment of the influence of a challenging adhesive restoration (submitted or not to thermocycling) on ​​the diffusion of H_2_O_2_ released from an in-office whitening gel and the possible adverse effects of different concentrations of this molecule on pulp cells.

This sequential thermal variation technique can promote expansion and shrinkage of the restorative material, resulting in constant stress at the tooth-adhesive restoration-bonding interface ^18^. Also, water absorption during thermocycling ^19^ and the storage of restored specimens in a humid environment can also contribute to hydrolytic degradation of the adhesive interface ^20^, favoring the diffusion of H_2_O_2_ from the bleaching gels ^21^. Thus, the higher concentration of this toxic molecule found in extracts of the RT/HD/HP group, besides significantly reducing the viability of MDPC-23 cells, also induced these pulp cells to increase the gene expression of inflammatory mediators COX-2 and TNF-α in relation to the other groups. It is important to highlight that the H_2_O_2_ quantification method used in the present investigation has limitations. The quantification was performed on the extracts that were applied to the MDPC-23 cells at the end of bleaching protocol, which may have resulted in some H_2_O_2_ degradation. In addition, this technique does not quantify other by-products, such as hydroxyl ion, that could have been involved in the trans-enamel and trans-dentinal toxic effects.

Both restored/whitened groups also exhibited an increase in the expression of IL-1β and IL-6 compared to the SD/HP group. Thus, it has been described that vascular/cellular events resulting from the increased expression of pro-inflammatory mediators in the pulp, which cause an increase in intra-pulp pressure and consequent mechanical stimulation of peripheral nerve fibers ^22^, result in local release of substance P (SP) and calcitonin gene-related peptide (CGRP) ^23^. In turn, these neuropeptides cause excitation of transmission neurons, triggering dental pain from damage caused to the pulp tissue ^23^. 

Markowitz (2010) suggested that inflammatory events in the pulp play an important role in dental sensitivity after in-office whitening, which has been frequently reported by patients undergoing this esthetic therapy ^24^. Thus, post-whitening tooth sensitivity seems to be directly related to the H_2_O_2_ concentration capable of reaching and causing damage to pulp cells ^24^. Randomized clinical trials have proposed the use of pre-emptive anti-inflammatory drugs, corticosteroids and/or analgesics to minimize the postoperative tooth sensitivity mediated by in-office whitening treatment with highly concentrated H_2_O_2_ gels ^25^. More recently, Meirelles et al. (2021) observed in a randomized clinical trial that adhesive restorations had no effect of tooth sensitivity when a 10% carbamide peroxide bleaching gel was used for two weeks. Thus, considering that results obtained in laboratory research cannot be directly extrapolated to clinical situations, data from this study indicate that clinicians should be careful when performing in-office whitening therapies in teeth with adhesive restorations. 

Nevertheless, several limitations related with this in vitro study should be considered, as the absence of pulpal pressure, the use of monolayer cells, and application of bleaching gels onto enamel/dentin discs from bovine teeth, as it can influence on H_2_O_2_ diffusion and pulpal cells response. Also, further in vivo and in vitro studies are needed to assess the influence of diverse dental materials, as well as the size and depth of restored dental cavities, on the diffusion of H_2_O_2_ and its effects on pulp cells. Besides that, the scientific evidence provided by this investigation denotes that minimizing the amount of free H_2_O_2_ capable of reaching the pulp chamber remains as the more secure option to prevent intense inflammatory pulp response and cell damage, as well as the undesired post-whitening tooth sensitivity. 

## Conclusion

Based on the methodology employed in this study, one can conclude that the tooth/adhesive restoration interface enhances the concentration of H_2_O_2_ capable of reaching the pulp space to cause toxic effects and to induce over-expression of pro-inflammatory mediators by pulp cells after performing a conventional in-office tooth whitening treatment. 
